# Association between lesion-specific fat attenuation index and high-risk plaque on coronary computed tomography angiography

**DOI:** 10.3389/fcvm.2026.1979906

**Published:** 2026-09-30

**Authors:** Zhikun Chen, Junmei Zhao, Yayun Liu, Ya Ma, Shitong Yin, Chunlan Bai, Yazhao Sun, Meiyu Zhang

**Affiliations:** 1 Department of Medical Imaging, Hebei Cangzhou Hospital of Integrated Traditional Chinese and Western Medicine, Cangzhou, Hebei, China; 2 Department of Neurology, Hebei Cangzhou Hospital of Integrated Traditional Chinese and Western Medicine, Cangzhou, Hebei, China; 3 Department of Cardiology, Cangzhou People’s Hospital, Cangzhou, Hebei, China; 4 Department of Ultrasound, Cangzhou People’s Hospital, Cangzhou, Hebei, China; 5 Department of Traditional Chinese Medicine, Hebei Cangzhou Hospital of Integrated Traditional Chinese and Western Medicine, Cangzhou, Hebei, China

**Keywords:** coronary atherosclerosis, coronary computed tomography angiography, fat attenuation index, high-risk plaque, pericoronary adipose tissue

## Abstract

**Objective:**

To investigate the association between lesion-specific fat attenuation index (FAI) derived from coronary computed tomography angiography (CCTA) and CCTA-defined high-risk plaque (HRP).

**Methods:**

A total of 560 patients undergoing CCTA for the first time who had at least one evaluable coronary atherosclerotic lesion were consecutively included. One target lesion was selected for each patient. Four CCTA-derived high-risk plaque features were assessed, including low-attenuation plaque, positive remodeling, spotty calcification, and the napkin-ring sign. Lesions exhibiting at least two of these features were classified as CCTA-defined HRP, and lesion-specific FAI was quantified from the pericoronary adipose tissue surrounding the target lesion and expressed in Hounsfield units (HU). Binary logistic regression was used to evaluate the association between lesion-specific FAI and CCTA-defined HRP. Restricted cubic spline analysis was performed to characterize the shape of the association, and subgroup and sensitivity analyses were conducted to evaluate the robustness of the findings.

**Results:**

Lesion-specific FAI was significantly higher in patients with CCTA-defined HRP than in those without HRP (−76.5 ± 4.8 HU vs. −81.0 ± 4.8 HU), with a mean difference of 4.47 HU [95% confidence interval (CI), 3.54–5.41 HU, *P* < 0.001] and a Cohen's *d* of 0.93. In the fully adjusted model, each 5-HU increase in lesion-specific FAI was associated with higher odds of CCTA-defined HRP [odds ratio (OR), 2.03; 95% CI, 1.52–2.69; *P* < 0.001]. Restricted cubic spline analysis demonstrated a significant overall association between lesion-specific FAI and CCTA-defined HRP (*P* for overall <0.001), with no significant evidence of nonlinearity (*P* for nonlinearity = 0.702). The association remained consistent across the examined subgroups, with no significant interactions, and the findings remained robust in sensitivity analyses.

**Conclusions:**

Higher lesion-specific FAI was independently associated with CCTA-defined HRP, with higher odds of HRP across increasing FAI values. The association remained consistent across subgroup and sensitivity analyses. Lesion-specific FAI may provide complementary information for characterizing CCTA-defined high-risk plaque and assessing the local perilesional environment. Further prospective studies with invasive imaging or longitudinal clinical outcomes are needed to determine its potential incremental value.

## Introduction

The clinical significance of coronary atherosclerotic plaques is not determined solely by the degree of luminal stenosis. Coronary computed tomography angiography (CCTA) enables simultaneous assessment of coronary stenosis and high-risk plaque (HRP) features, including low-attenuation plaque, positive remodeling, spotty calcification, and the napkin-ring sign. Ferencik et al. (1) demonstrated in the PROMISE trial that HRP features identified by CCTA were associated with subsequent cardiovascular events. The SCOT-HEART study further showed that low-attenuation plaque burden was strongly associated with subsequent myocardial infarction (2). These findings indicate that plaque characteristics provide clinically relevant information beyond the severity of coronary stenosis.

Inflammation plays an important role in atherosclerotic plaque progression and destabilization. Antonopoulos et al. (3) showed that coronary inflammation alters the phenotype and computed tomography (CT) attenuation of perivascular adipose tissue and introduced the fat attenuation index (FAI) as a noninvasive imaging marker of coronary inflammation. The CRISP-CT study subsequently demonstrated that FAI provided prognostic information beyond conventional cardiovascular risk factors and coronary anatomical characteristics (4). More recently, the ORFAN study further supported the value of CT-based coronary inflammation assessment for cardiovascular prognostic assessment (5).

Because coronary inflammation may vary locally along the coronary tree, lesion-specific FAI may more directly reflect the inflammatory milieu surrounding an individual plaque. Yuki et al. (6), using combined CCTA and optical coherence tomography, demonstrated a close relationship between pericoronary fat attenuation and plaque vulnerability. Liu et al. (7) further reported that higher lesion-specific FAI was associated with HRP and adverse cardiovascular events. Recent studies have also demonstrated associations between perivascular inflammation and HRP characteristics (8, 9). However, the independent association between lesion-specific FAI and HRP, particularly its pattern across the FAI range, remains incompletely characterized. Therefore, this study aimed to investigate the association between lesion-specific FAI and HRP and to further characterize the shape of this association and its consistency across clinically relevant subgroups.

## Materials and methods

### Study design and participants

This was a single-center retrospective study that consecutively included patients who underwent CCTA for the first time at Hebei Cangzhou Hospital of Integrated Traditional Chinese and Western Medicine between January 2024 and January 2026. The inclusion criteria were as follows: (1) age ≥18 years; (2) first-time CCTA examination; (3) at least one evaluable coronary atherosclerotic lesion identified on CCTA; and (4) complete clinical and laboratory data. The exclusion criteria were as follows: (1) a known history of coronary artery disease (CAD); (2) poor CCTA image quality due to marked motion artifacts, stair-step artifacts, or other artifacts that precluded reliable assessment of coronary plaque characteristics or lesion-specific FAI; and (3) missing key clinical or laboratory data. A total of 560 patients were ultimately included, of whom 136 had HRP and 424 did not. The study was approved by the Ethics Committee of Hebei Cangzhou Hospital of Integrated Traditional Chinese and Western Medicine (approval No. 2026-KY083). All patients provided written informed consent at the time of clinical evaluation and agreed that their anonymized clinical and imaging data could be used for subsequent medical research.

### Clinical data collection

Baseline clinical data were obtained from the electronic medical records, including age, sex, body mass index (BMI), current smoking status, hypertension, diabetes mellitus, statin use before CCTA, and clinical presentation at the time of CCTA. Clinical presentation was categorized as symptomatic or asymptomatic according to the presence or absence of suspected ischemic symptoms, including chest pain or chest discomfort, documented at the time of CCTA evaluation. Laboratory measurements included total cholesterol (TC), triglycerides (TG), high-density lipoprotein cholesterol (HDL-C), and low-density lipoprotein cholesterol (LDL-C), using the most recent results available before CCTA. Hypertension and diabetes mellitus were defined according to a documented medical history or current use of corresponding medications. Current smoking was defined as active smoking at the time of CCTA, and statin use was defined as ongoing statin therapy before CCTA.

### CCTA image analysis

CCTA images were transferred to a dedicated post-processing workstation for coronary artery analysis. Lesion-specific FAI was quantified using an artificial intelligence-assisted coronary analysis platform (CoronaryDoc FAI Analysis Tool, version V7.05, ShuKun Technology, Beijing, China). The software automatically performed coronary artery centerline reconstruction, vessel wall identification, and segmentation of PCAT surrounding the target lesion. The automatically generated contours were visually reviewed and manually adjusted when necessary to ensure accurate delineation of the target lesion region. The target lesion vessel was recorded as the left anterior descending artery (LAD), right coronary artery (RCA), or left circumflex artery (LCX), and the degree of luminal diameter stenosis was categorized as <50%, 50%–69%, or ≥70%. One target lesion was selected for each patient. In patients with multiple evaluable coronary atherosclerotic lesions, the lesion with the greatest degree of luminal stenosis was selected as the target lesion. Four HRP features were assessed, including low-attenuation plaque, positive remodeling, spotty calcification, and the napkin-ring sign. Low-attenuation plaque was defined as a plaque containing an area with CT attenuation <30 Hounsfield units (HU). Positive remodeling was defined as a remodeling index ≥1.1. Spotty calcification was defined as focal calcification within the plaque with a maximum diameter <3 mm. The napkin-ring sign was defined as a central low-attenuation region surrounded by a rim of higher attenuation. A target lesion exhibiting at least two of these features was classified as HRP, whereas lesions with fewer than two features were classified as non-HRP. PCAT was defined as adipose tissue with CT attenuation values ranging from −190 to −30 HU within a radial distance from the outer vessel wall equivalent to the diameter of the corresponding coronary artery. The lesion-specific FAI was calculated as the mean CT attenuation value of all adipose tissue voxels within the PCAT region surrounding the target lesion and was expressed in HU.

### Statistical analysis

Continuous variables were expressed as mean ± standard deviation or median with interquartile range, according to their distribution. Normally distributed continuous variables were compared using the independent-samples *t* test, whereas non-normally distributed variables were compared using the Mann–Whitney *U* test. Categorical variables were expressed as counts and percentages and compared using the *χ*^2^ test. For lesion-specific FAI, the between-group difference was additionally reported as the mean difference with its 95% confidence interval (CI), and Cohen's *d* was calculated to quantify the effect size.

Binary logistic regression analysis was performed to evaluate the association between lesion-specific FAI and CCTA-defined HRP, with odds ratios (ORs) and 95% CIs reported per 5-HU increase in FAI. Model 1 was unadjusted. Model 2 was adjusted for age and sex. Model 3 was further adjusted for BMI, current smoking, hypertension, diabetes mellitus, LDL-C, statin use, and degree of target lesion stenosis. These covariates were selected *a priori* based on their established associations with coronary atherosclerosis, plaque vulnerability, and pericoronary adipose tissue characteristics, and were considered potential confounders of the association between lesion-specific FAI and HRP. Restricted cubic spline analysis was used to characterize the shape of the association between lesion-specific FAI and HRP. The median FAI value was used as the reference, with four knots placed at the 5th, 35th, 65th, and 95th percentiles. Overall association and nonlinearity were assessed. Subgroup analyses were performed according to age, sex, current smoking, diabetes mellitus, statin use, and degree of target lesion stenosis, and interaction tests were conducted to assess potential effect modification. Sensitivity analyses were performed by separately excluding patients with target lesion stenosis ≥70% and those who had used statins before CCTA. The fully adjusted model was refitted in both sensitivity analyses, with statin use omitted from the analysis excluding statin users. All tests were two-sided, and a *P* value <0.05 was considered statistically significant. All statistical analyses were performed using R software (version 4.4.3, R Foundation for Statistical Computing, Vienna, Austria).

## Results

### Clinical and coronary lesion characteristics

A total of 560 patients were included, of whom 24.3% had CCTA-defined HRP. Compared with the non-HRP group, patients in the HRP group were older and had higher proportions of males, hypertension, and diabetes mellitus, as well as higher LDL-C levels. In contrast, no statistically significant differences were observed between the two groups in TC, TG, HDL-C, BMI, current smoking status, or statin use. Clinical presentation did not differ significantly between the HRP and non-HRP groups. The distribution of target lesion vessels was comparable between the two groups. However, the HRP group had more severe target lesion stenosis and higher lesion-specific FAI values (Table 1).

**Table 1:** Clinical and CCTA characteristics according to CCTA-defined high-risk plaque status.

Characteristic	Overall *N* = 560	Non-HRP *N* = 424	HRP *N* = 136	*P* value
Age, years	61.0 ± 8.6	59.7 ± 8.0	64.9 ± 9.1	<0.001
Male sex, *n* (%)	322 (57.5%)	232 (54.7%)	90 (66.2%)	0.019
BMI, kg/m²	25.6 ± 2.7	25.5 ± 2.6	25.8 ± 2.8	0.194
Current smoking, *n* (%)	158 (28.2%)	113 (26.7%)	45 (33.1%)	0.147
Hypertension, *n* (%)	247 (44.1%)	176 (41.5%)	71 (52.2%)	0.029
Diabetes mellitus, *n* (%)	107 (19.1%)	67 (15.8%)	40 (29.4%)	<0.001
Total cholesterol, mmol/L	4.95 ± 0.70	4.93 ± 0.69	5.04 ± 0.74	0.130
Triglycerides, mmol/L	1.42 (1.07, 1.86)	1.43 (1.12, 1.87)	1.41 (1.04, 1.79)	0.239
HDL-C, mmol/L	1.24 ± 0.21	1.24 ± 0.22	1.21 ± 0.20	0.104
LDL-C, mmol/L	3.01 ± 0.59	2.96 ± 0.58	3.16 ± 0.62	<0.001
Statin use, *n* (%)	56 (10.0%)	41 (9.7%)	15 (11.0%)	0.646
Clinical presentation, *n* (%)				0.641
Symptomatic	260 (46.4%)	194 (45.8%)	66 (48.5%)	
Asymptomatic	300 (53.6%)	230 (54.2%)	70 (51.5%)	
Target lesion vessel				0.353
LAD	295 (52.7%)	221 (52.1%)	74 (54.4%)	
RCA	164 (29.3%)	121 (28.5%)	43 (31.6%)	
LCX	101 (18.0%)	82 (19.3%)	19 (14.0%)	
Diameter stenosis				<0.001
<50%	280 (50.0%)	241 (56.8%)	39 (28.7%)	
50%–69%	168 (30.0%)	129 (30.4%)	39 (28.7%)	
≥70%	112 (20.0%)	54 (12.7%)	58 (42.6%)	
Lesion-specific FAI, HU	−79.9 ± 5.2	−81.0 ± 4.8	−76.5 ± 4.8	<0.001
Low-attenuation plaque, *n* (%)	151 (27.0%)	47 (11.1%)	104 (76.5%)	
Positive remodeling, *n* (%)	136 (24.3%)	43 (10.1%)	93 (68.4%)	
Spotty calcification, *n* (%)	107 (19.1%)	28 (6.6%)	79 (58.1%)	
Napkin-ring sign, *n* (%)	50 (8.9%)	12 (2.8%)	38 (27.9%)	

BMI, body mass index; CCTA, coronary computed tomography angiography; FAI, fat attenuation index; HDL-C, high-density lipoprotein cholesterol; HRP, high-risk plaque; HU, Hounsfield unit; LAD, left anterior descending artery; LCX, left circumflex artery; LDL-C, low-density lipoprotein cholesterol; RCA, right coronary artery.

### Lesion-specific FAI according to high-risk plaque status

Lesion-specific FAI was significantly higher (i.e., less negative) in the HRP group than in the non-HRP group (−76.5 ± 4.8 HU vs. −81.0 ± 4.8 HU). The mean between-group difference in FAI was 4.47 HU (95% CI, 3.54–5.41 HU; *P* < 0.001), with a Cohen's *d* of 0.93, indicating a large effect size.

### Multivariable association between lesion-specific FAI and HRP

To evaluate the independent association between lesion-specific FAI and HRP, we constructed sequentially adjusted logistic regression models. In the unadjusted model, each 5-HU increase in lesion-specific FAI was associated with higher odds of HRP (OR, 2.66; 95% CI, 2.10–3.38; *P* < 0.001). After adjustment for age and sex, the association was attenuated but remained statistically significant (OR, 2.31; 95% CI, 1.79–2.98; *P* < 0.001). In the fully adjusted model, which further included BMI, current smoking, hypertension, diabetes mellitus, LDL-C, statin use, and degree of target lesion stenosis, lesion-specific FAI remained independently associated with HRP (OR, 2.03; 95% CI, 1.52–2.69; *P* < 0.001) (Table 2).

**Table 2:** Association between lesion-specific fat attenuation index and CCTA-defined high-risk plaque.

Model	OR per 5-HU Increase in FAI (95% CI)	*P* Value
Model 1	2.66 (2.10–3.38)	<0.001
Model 2	2.31 (1.79–2.98)	<0.001
Model 3	2.03 (1.52–2.69)	<0.001

BMI, body mass index; CI, confidence interval; FAI, fat attenuation index; HRP, high-risk plaque; HU, Hounsfield unit; LDL-C, low-density lipoprotein cholesterol; OR, odds ratio.

ORs are reported per 5-HU increase in lesion-specific FAI. Model 1 was unadjusted; Model 2 was adjusted for age and sex; Model 3 was additionally adjusted for BMI, current smoking, hypertension, diabetes mellitus, LDL-C, statin use, and degree of target lesion stenosis.

### Restricted cubic spline analysis of the association between lesion-specific FAI and HRP

Restricted cubic spline analysis demonstrated a significant overall association between lesion-specific FAI and HRP across all three models (all *P* for overall < 0.001). No significant evidence of nonlinearity was observed in the unadjusted model, the age- and sex-adjusted model, or the fully adjusted model (*P* for nonlinearity = 0.909, 0.872, and 0.702, respectively). Using the median lesion-specific FAI value of −80.0 HU as the reference, the odds ratio for HRP increased progressively with increasing lesion-specific FAI, and this association remained evident in the fully adjusted model. Restricted cubic splines were fitted using four knots placed at the 5th, 35th, 65th, and 95th percentiles of lesion-specific FAI, corresponding to −88.8, −81.9, −77.7, and −71.8 HU, respectively (Figure 1).

**Figure 1:**
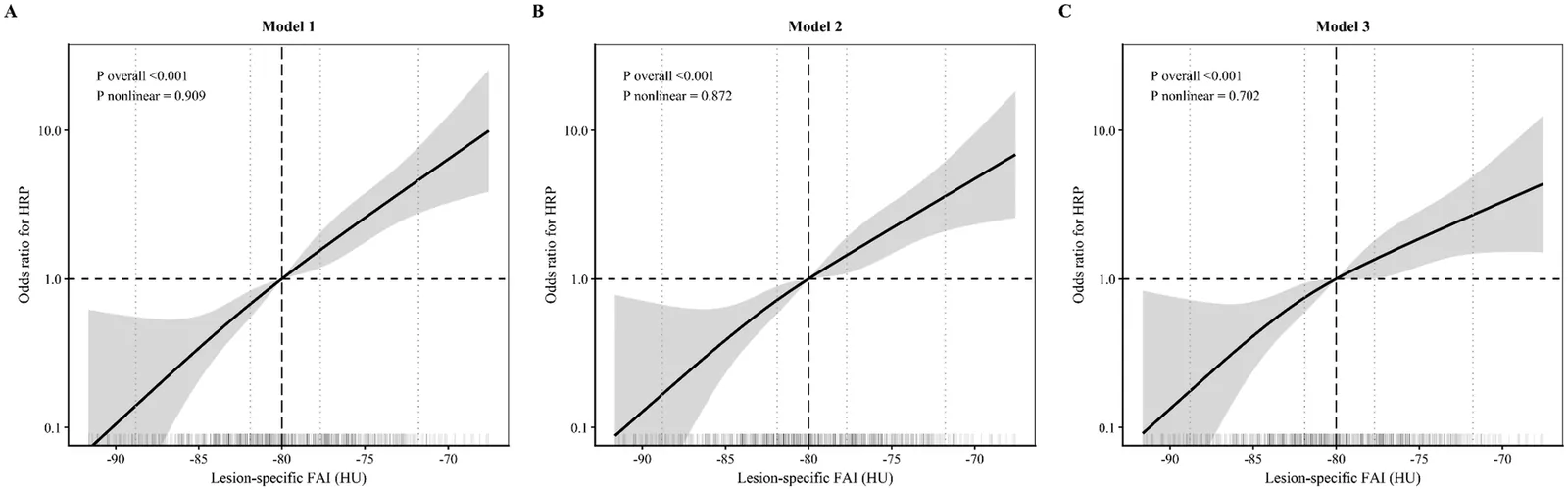
Restricted cubic spline analysis of the association between lesion-specific FAI and CCTA-defined HRP. **(A)** Model 1, unadjusted; **(B)** model 2, adjusted for age and sex; and **(C)** Model 3, additionally adjusted for BMI, current smoking, hypertension, diabetes mellitus, LDL-C, statin use, and degree of target lesion stenosis. BMI, body mass index; CI, confidence interval; FAI, fat attenuation index; HRP, high-risk plaque; HU, Hounsfield unit; LDL-C, low-density lipoprotein cholesterol; OR, odds ratio.

### Subgroup analyses

Across subgroups stratified by age, sex, current smoking, diabetes mellitus, statin use, and degree of target lesion stenosis, the positive association between lesion-specific FAI and HRP remained generally consistent, with statistically significant associations observed in all subgroups. No significant interactions were identified across the subgroups, suggesting no evidence of effect modification by these factors (Figure 2).

**Figure 2:**
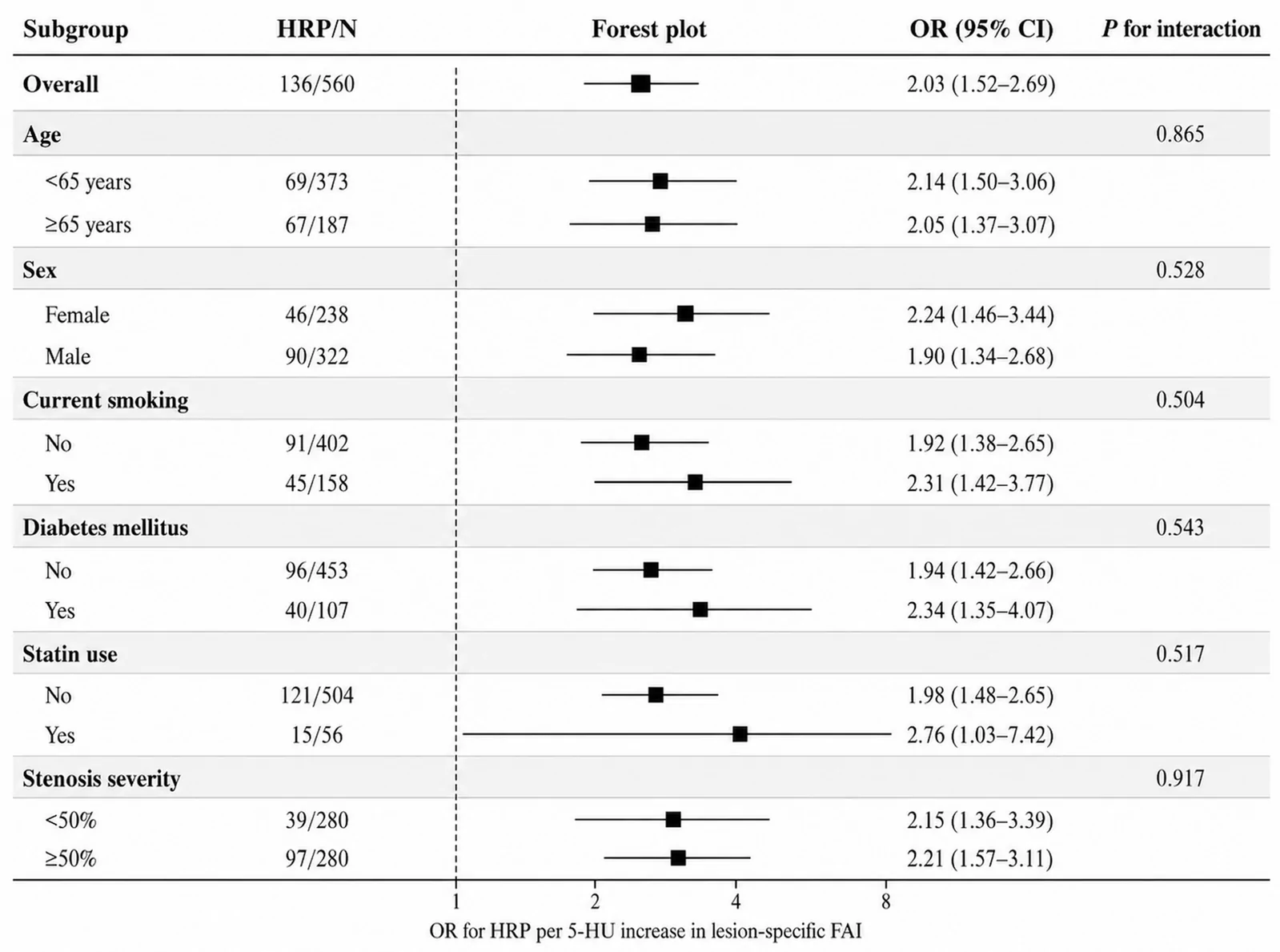
Subgroup analyses of the association between lesion-specific FAI and CCTA-defined HRP. Subgroup analyses were adjusted for age, sex, BMI, current smoking, hypertension, diabetes mellitus, LDL-C, statin use, and degree of target lesion stenosis, except for the variable used for stratification. BMI, body mass index; CI, confidence interval; FAI, fat attenuation index; HRP, high-risk plaque; HU, Hounsfield unit; LDL-C, low-density lipoprotein cholesterol; OR, odds ratio.

### Sensitivity analyses

We performed two sensitivity analyses to assess the robustness of the main findings. After excluding patients with target lesion stenosis ≥70%, lesion-specific FAI remained significantly associated with HRP (OR, 1.99; 95% CI, 1.42–2.79; *P* < 0.001). Similarly, after excluding patients who had used statins before CCTA, the association remained stable (OR, 2.00; 95% CI, 1.49–2.70; *P* < 0.001). The results of both sensitivity analyses were consistent with the main analysis, further supporting the robustness of the association between lesion-specific FAI and HRP (Table 3).

**Table 3:** Sensitivity analyses of the association between lesion-specific fat attenuation index and high-risk plaque.

Sensitivity analysis	*N* (HRP cases)	OR (95% CI)	*P* Value
Excluding patients with target lesion stenosis ≥70%	448 (78)	1.99 (1.42–2.79)	<0.001
Excluding statin users before CCTA	504 (121)	2.00 (1.49–2.70)	<0.001

CCTA, coronary computed tomography angiography; CI, confidence interval; FAI, fat attenuation index; HRP, high-risk plaque; HU, Hounsfield unit; OR, odds ratio.

ORs are reported per 5-HU increase in lesion-specific FAI and were estimated using the fully adjusted model. Statin use was omitted from the model in the analysis excluding statin users.

## Discussion

Lesion-specific FAI was substantially higher in lesions with CCTA-defined HRP than in those without HRP. In the fully adjusted model, each 5-HU increase in lesion-specific FAI was associated with approximately twofold higher odds of HRP. The association increased progressively across the observed FAI range without evidence of a clear nonlinear threshold and remained stable across clinically relevant subgroups and sensitivity analyses. These findings support a close relationship between the attenuation characteristics of adipose tissue surrounding an individual coronary lesion and the high-risk morphological features identified by CCTA of that same lesion. This relationship persisted after accounting for conventional cardiovascular risk factors and the severity of luminal stenosis, indicating that the association is not fully explained by anatomical stenosis alone.

Previous lesion-level studies have reported similar observations. Goeller et al. found higher PCAT attenuation around culprit and high-risk plaques in patients with acute coronary syndrome (10). Ma et al. showed that focal PCAT attenuation was associated with plaque presence, plaque type, and stenosis severity, supporting the use of measurements centered on individual coronary lesions (11). Sun et al. linked higher FAI to imaging-based markers of plaque vulnerability and local immune-inflammatory activation in patients with non-ST-segment elevation acute coronary syndrome (12), while Zhang et al. reported associations between lesion-specific FAI, plaque characteristics, and stenosis severity (13). Recent reviews have further summarized the emerging role of PCAT inflammation and FAI derived from advanced CCTA as complementary imaging biomarkers for biological characterization of coronary atherosclerosis beyond conventional anatomical assessment (14). The present study extends these observations to patients undergoing first-time CCTA and shows that the association between lesion-specific FAI and CCTA-defined HRP remains evident after lesion stenosis is taken into account. Lesion-specific FAI may therefore reflect features of the local lesion environment that are not captured by anatomical disease severity.

Evidence from culprit-lesion and multimodality imaging studies further supports this relationship. Kuneman et al. showed that lesions that subsequently became culprit lesions already exhibited higher local PCAT attenuation on antecedent CCTA (15). The ICONIC study found only modest absolute differences in PCAT attenuation between patients with future acute coronary syndromes and matched controls, but a residual association remained after accounting for plaque burden and high-risk plaque characteristics (16). Li et al. reported higher plaque-specific FAI in culprit than in nonculprit lesions (17). Studies combining CCTA with optical coherence tomography have suggested that higher PCAT attenuation is associated with imaging and pathological features related to plaque vulnerability, including lipid-rich plaque, macrophage accumulation, and thin-cap fibroatheroma (18). Choi et al. further demonstrated that CT-based assessment of coronary inflammation provides information that is not fully overlapping with plaque vulnerability or fractional flow reserve (19). HRP morphology and lesion-specific FAI may therefore describe different dimensions of the same lesion: structural plaque phenotype and the surrounding biological milieu.

Serial imaging studies suggest that PCAT attenuation is also linked to plaque evolution. Changes in PCAT attenuation have been associated with changes in noncalcified plaque burden, while higher baseline PCAT attenuation has been related to subsequent alterations in noncalcified plaque composition (20, 21). Long-term serial CCTA studies have similarly linked greater pericoronary inflammation with adverse plaque progression (22). However, evidence regarding long-term clinical outcomes is not entirely consistent. Higher PCAT attenuation has been associated with cardiovascular events in patients with type 2 diabetes, whereas other long-term studies have not shown an independent association with major adverse cardiovascular events after comprehensive adjustment (23, 24). Treatment studies also indicate that PCAT attenuation is potentially dynamic. Statin therapy has been associated with reductions in lesion-level PCAT attenuation (25), whereas low-dose colchicine did not significantly alter PCAT attenuation in an imaging substudy of the LoDoCo2 trial (26). Post-mortem histopathological data further suggest that FAI should not be interpreted as a direct measure of inflammatory-cell infiltration (27). A recent systematic review and meta-analysis nevertheless found an overall association of higher PCAT attenuation with HRP and adverse cardiovascular outcomes, while also identifying substantial heterogeneity across measurement approaches (28). The absence of a clear nonlinear threshold in the present study also argues against deriving a universal FAI cutoff from these data. Retaining FAI as a continuous variable may preserve more lesion-level information than an arbitrary high-vs.-low classification.

The absolute value of PCAT attenuation is sensitive to image acquisition and reconstruction. Differences in coronary territory and tube voltage can alter measured attenuation (29), and phantom studies have demonstrated additional effects of iterative reconstruction (30). More recent photon-counting CT work has shown that vascular attenuation, virtual monoenergetic level, and reconstruction kernel can also influence PCAT measurements (31). These technical dependencies are particularly relevant to lesion-specific analysis because the measurement is performed over a relatively localized region. Standardization of acquisition, reconstruction, and post-processing will therefore be necessary before lesion-specific FAI values can be compared reliably across scanner platforms and centers.

Several limitations should be acknowledged. First, this was a single-center retrospective study and was therefore subject to potential selection bias and residual confounding. Second, only one target lesion was analyzed per patient, and the lesion with the greatest degree of luminal stenosis was selected in patients with multiple lesions. The findings therefore reflect the local characteristics of the selected lesion rather than the overall plaque and inflammatory burden of the coronary tree. This selection strategy may have preferentially included more advanced lesions and may limit the generalizability of the findings to the overall coronary atherosclerotic burden. Third, HRP was defined based on CCTA-derived morphological features without validation by optical coherence tomography, intravascular ultrasound, histopathology, or longitudinal clinical outcomes. Therefore, the findings should be interpreted as an association between lesion-specific FAI and CCTA-defined HRP rather than direct evidence of histological plaque vulnerability or future cardiovascular risk prediction. Fourth, lesion-specific FAI and HRP assessment were obtained from the same CCTA examination and target lesion region. Therefore, the observed association may also be influenced by imaging-related factors, including image quality, contrast enhancement, plaque delineation, reconstruction parameters, and local blooming or partial-volume effects. In addition, formal intra- and interobserver reproducibility analyses for lesion-specific FAI measurements were not performed in the present study, and further validation of measurement reproducibility is warranted. Further studies using standardized imaging protocols and independent validation are needed to confirm these findings. Finally, longitudinal imaging and clinical outcome data were unavailable, precluding assessment of temporal relationships, plaque progression, and the prognostic value of lesion-specific FAI. Although clinical presentation was evaluated and showed no significant difference between groups, unmeasured aspects of clinical status may still have contributed to residual confounding.

## Conclusion

Higher lesion-specific FAI was independently associated with CCTA-defined HRP, with higher odds of HRP across increasing FAI values. The association remained consistent across subgroup and sensitivity analyses. Lesion-specific FAI may complement CCTA assessment of luminal stenosis and plaque morphology by providing information on the local perilesional environment. However, these findings should be interpreted as an association with CCTA-defined HRP rather than direct evidence of histological plaque vulnerability or clinical event prediction. Prospective multicenter studies are needed to confirm these findings and assess the reproducibility and potential incremental value of lesion-specific FAI.

## Data Availability

The original contributions presented in the study are included in the article/Supplementary Material, further inquiries can be directed to the corresponding author.
